# Hepatic hydrogen sulfide levels are reduced in mouse model of Hutchinson-Gilford progeria syndrome

**DOI:** 10.18632/aging.204835

**Published:** 2023-06-23

**Authors:** Stephen E. Wilkie, Diana E. Marcu, Roderick N. Carter, Nicholas M. Morton, Susana Gonzalo, Colin Selman

**Affiliations:** 1 Glasgow Ageing Research Network (GARNER), School of Biodiversity, One Health and Veterinary Medicine, College of Medical, Veterinary and Life Sciences, University of Glasgow, Glasgow G12 8QQ, UK; 2 Molecular Metabolism Group, University/BHF Centre for Cardiovascular Sciences, Queens Medical Research Institute, University of Edinburgh, Edinburgh EH16 4TJ, UK; 3 Department of Biochemistry and Molecular Biology, Edward A. Doisy Research Center, Saint Louis University School of Medicine, MO 63104, USA; 4 Division of Molecular Metabolism, Department of Medical Biochemistry and Biophysics, Karolinska Institute, Solna 171 65, Sweden

**Keywords:** progeria, hydrogen sulfide, high-fat diet, ageing, lamin A

## Abstract

Hutchinson-Gilford progeria syndrome (HGPS) is a rare human disease characterised by accelerated biological ageing. Current treatments are limited, and most patients die before 15 years of age. Hydrogen sulfide (H_2_S) is an important gaseous signalling molecule that it central to multiple cellular homeostasis mechanisms. Dysregulation of tissue H_2_S levels is thought to contribute to an ageing phenotype in many tissues across animal models. Whether H_2_S is altered in HGPS is unknown. We investigated hepatic H_2_S production capacity and transcript, protein and enzymatic activity of proteins that regulate hepatic H_2_S production and disposal in a mouse model of HGPS (G609G mice, mutated Lmna gene equivalent to a causative mutation in HGPS patients). G609G mice were maintained on either regular chow (RC) or high fat diet (HFD), as HFD has been previously shown to significantly extend lifespan of G609G mice, and compared to wild type (WT) mice maintained on RC. RC fed G609G mice had significantly reduced hepatic H_2_S production capacity relative to WT mice, with a compensatory elevation in mRNA transcripts associated with several H_2_S production enzymes, including cystathionine-γ-lyase (CSE). H_2_S levels and CSE protein were partially rescued in HFD fed G609G mice. As current treatments for patients with HGPS have failed to confer significant improvements to symptoms or longevity, the need for novel therapeutic targets is acute and the regulation of H_2_S through dietary or pharmacological means may be a promising new avenue for research.

## INTRODUCTION

Progeroid syndromes are a set of genetic disorders characterized by a shortened lifespan and early onset phenotypic alterations typically associated with advanced age such as hair loss, frailty, and atherosclerosis [1]. Consequently, studies of progeroid syndromes in both humans and in model organisms have provided valuable insight into the ageing process [2–4]. Progeria syndromes are extremely rare in humans, the most common is Hutchinson-Gilford Progeria Syndrome (HGPS), which has a prevalence of 1 in 20 million [5]. HGPS is a laminopathy, one of a set of diseases caused by mutations in the lamin A (LMNA) gene or genes that encode for proteins involved in lamin processing [6, 7]. Specific mutations activate a cryptic splice site, resulting in the production of a permanently farnesylated and methylated lamin-A isoform named progerin. Progerin disrupts the nuclear membrane by binding to lamin-A, stimulating senescence, and is associated with dramatically reduced lifespan [8]. Progerin also accumulates in tissues during normative ageing due to spontaneous activation of the cryptic splice site in HGPS, which further suggests that normative ageing and progeroid syndromes may share some common molecular processes [9].

The most common mutations in HGPS is the point mutation c.1824C>T;p.Gly608Gly in the human *LMNA* gene. A mouse model carrying the equivalent 1827C>T;p.Gly609Gly mutation in the *Lmna* gene has been developed and is known as Lmna^G609G+/G609G+^ or simply G609G [10]. G609G mice capture much of the human HGPS disease phenotype [11–13, 10], although some aspects of the human disease are not present in G609G mice. For instance, the leading cause of death (>90%) in HGPS patients is myocardial infarction or other cardiovascular pathologies such as stroke [14]. In the G609G mouse model the major cause of death is cachexia and starvation [15]. Diet has recently emerged as an intervention capable of modulating the progeroid phenotype in mice, with methionine restriction extending mean and median lifespan in G609G mice by approximately 20% [16, 17]. Methionine restriction is also reported to elevate tissue hydrogen sulfide (H_2_S) levels in various model systems [18], although H_2_S levels in G609G mice under methionine restriction has not yet been measured. G609G mice fed a high-fat diet (HFD) had an average lifespan of 193 days compared to 110 days on regular chow (RC) [15]. Surprisingly, G609G HFD mice had significantly lower serum glucose and plasma insulin levels compared to WT HFD mice [15], and improved glucose tolerance compared to G609G RC fed mice [15]. HFD feeding in G609G mice also elicited the onset of previously unseen pathologies in mice that closer replicate the phenotype of HGPS patients, including full body alopecia, skeletal dysplasia and aortic wall stiffening [15]. As such, the application of HFD in G609G mice may be considered as therapeutic (as is greatly extends lifespan compared to RC controls) but also may prove useful if employed as standard animal care conditions for G609G to more fully recapitulate the disease phenotype seen in patients with HGPS.

Therapeutic treatments for patients with progeroid diseases are lacking, with an average life expectancy typically less than 15 years [19]. Current treatments include farnesyltransferase inhibitors, rapamycin analogues, sulforaphane and vitamin D analogues, all of which all have clear impacts on cellular processes associated with HGPS but have yet to translate to substantial improvements to patient lifespan or incidence of comorbidities [19]. H_2_S has recently emerged as a potential effector of various longevity boosting interventions, and elevated tissue H_2_S levels are associated with long lived mutant mouse models [20–24].

While no studies to date have investigated the role of H_2_S in HGPS, there is overlap between H_2_S signalling and the mechanisms that underpin rapamycin, sulforaphane, and vitamin D treatment, as discussed in depth in our recent review [25]. Studies in mice have shown that *ex vivo* H_2_S production is negatively affected across tissues under HFD feeding, with a reduction in the protein levels of H_2_S-generating enzyme cystathionine-γ-lyase (CSE) [26]. Additionally, intraperitoneal injection of NaHS, a H_2_S donor compound, mitigated against markers of renal injury caused by HFD-induced obesity in mice [27]. These anti-obesity effects appear to be, in part, modulated through interaction between H_2_S and the mammalian target of rapamycin pathway [28], but also via the maintenance of mitochondrial import homeostasis and metabolic activity [29]. These data suggest that the deleterious effects of HFD in mice are associated with a reduced H_2_S production. However, given the unexpected benefits of HFD to lifespan in G609G, a greater understanding of H_2_S metabolism within the unique context of G609G mice on HFD may help further our understanding of the role of diet and H_2_S in progeria.

## RESULTS

**Figure 1:**
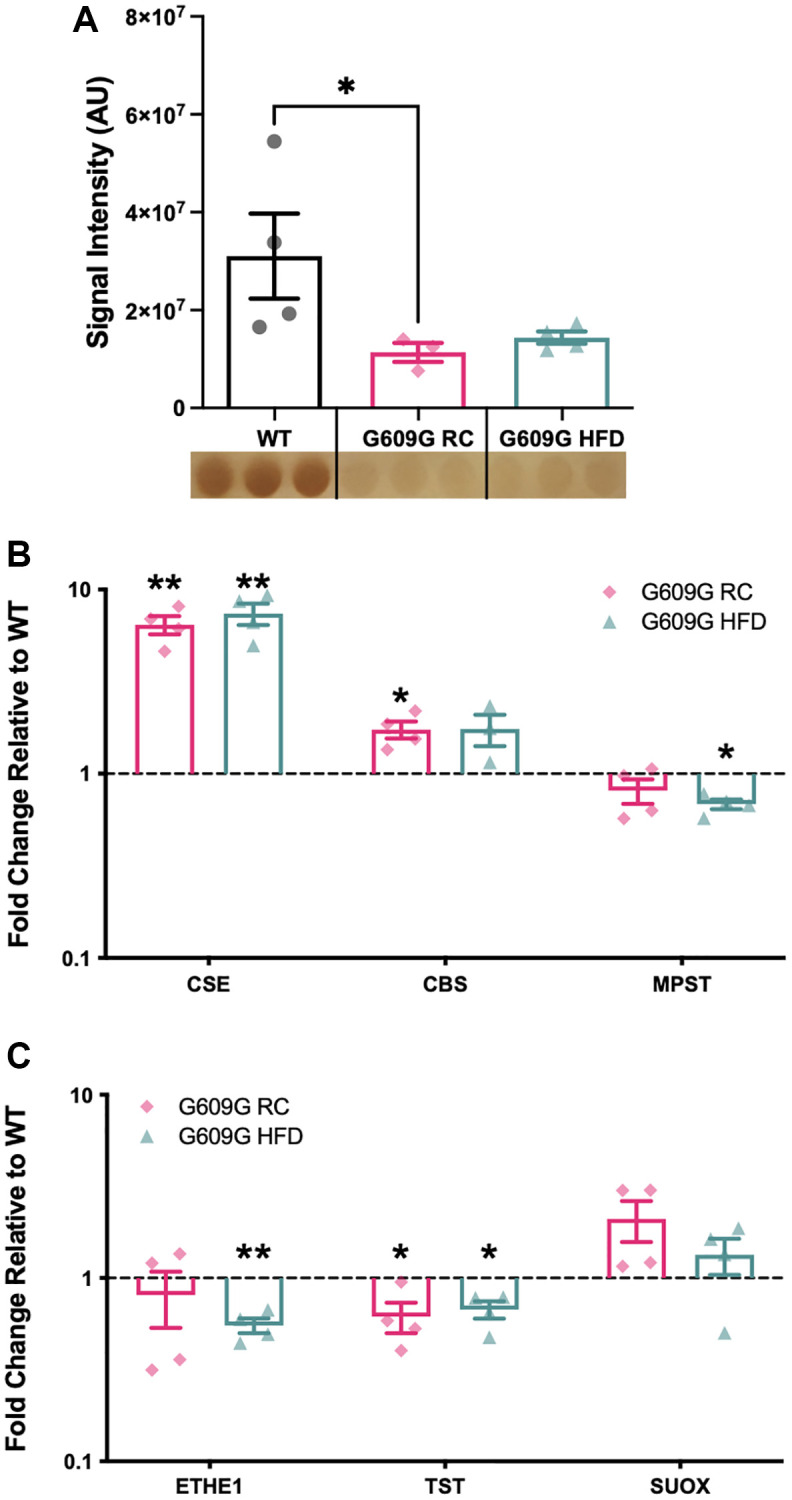
**Gene expression of selected H_2_S production and removal enzymes.** (**A**) Hepatic H_2_S production capacity assay assessed in protein lysates from WT, G609G RC and G609G HFD mice. Data quantified by densitometry analysis of lead acetate assay results. A representative image of the lead sulfide precipitate that form as the output of the lead acetate assay is shown beneath the plot. Darker precipitates indicate higher hepatic H_2_S producing enzymes *Cse*, *Cbs*, Mpst (**B**) and H_2_S disposal enzymes *Ethe1*, *Tst* and *Suox* (**C**) as measured by RT-qPCR. Relative expression values were calculated using the 2^−ΔΔCt^ method. WT data shown in black, G609G RC in pink, G609G in Green. Statistical significance determined by one-sample *t*-test comparing the fold changes to a theoretical mean of one. Grubbs outlier test with alpha = 0.05 was performed, no outliers removed. Bars show mean values with error bars representing standard error of the mean. Abbreviations: *Cse*: Cystathionine-beta-lyase; *Cbs*: Cystathionine-Beta-synthase; *Mpst*: 3-Mercaptopyruvate Sulfurtransferase; *Ethe1*: Ethylmalonic encephalopathy 1 protein; *Tst*: Thiosulfate Sulfurtransferase; *Suox*: Sulfite Oxidase. ^*^p < 0.05, ^**^p < 0.01.

Hepatic tissue protein homogenates were analysed by the lead acetate assay (Figure 1A). A greater than 50% reduction in H_2_S production capacity was found between regular-chow (RC) fed WT and G609G RC mice (*p* = 0.041), while a non-significant reduction of approximately 45% was found between WT and G609G HFD mice (*p* = 0.210). There was no significant difference in H_2_S production capacity between G609G mice on either diet (*p* > 0.999). Given the significant reduction in hepatic H_2_S production capacity in G609G RC mice compared to WT controls, we then investigated whether major components of the enzymatic H_2_S production and disposal processed were altered (Figure 1B and Figure 1C respectively). Transcript levels of *Cse* (also known as *Cth*) were found to be increased by almost 8-fold in G609G mice irrespective of diet (Figure 1B, *p* = 0.005 for RC and *p* = 0.007 for HFD). *Cbs* expression was almost doubled in G609G RC-fed mice relative to WT controls (*p* = 0.028), but comparable after HFD-feeding between genotypes (*p* = 0.558). *Mpst* transcript levels were not different between WT and G609G RC mice (*p* = 0.216) but were reduced by approximately 30% in G609G HFD mice relative to WT controls (*p* = 0.050). *Cse*, *Cbs*, and *Mpst* expression were unaffected by diet in G609G mice. We then examined expression levels of enzymes that regulate the oxidation and disposal of H_2_S. Ethylmalonic encephalopathy 1 protein (*Ethe1*) expression was significantly reduced in G609G HFD mice compared to WT mice (*p* = 0.003) but not different in G609G RC mice relative to WT mice (*p* = 0.525). Thiosulfate sulfurtransferase (*Tst*) expression was reduced in G609G mice relative to WT mice, irrespective of diet (*p* = 0.047 for RC and *p* = 0.027 for HFD). Finally, *Suox* expression was comparable between G609G and WT mice irrespective of diet (*p* = 0.128 for RC and *p* = 0.340 for HFD).

**Figure 2:**
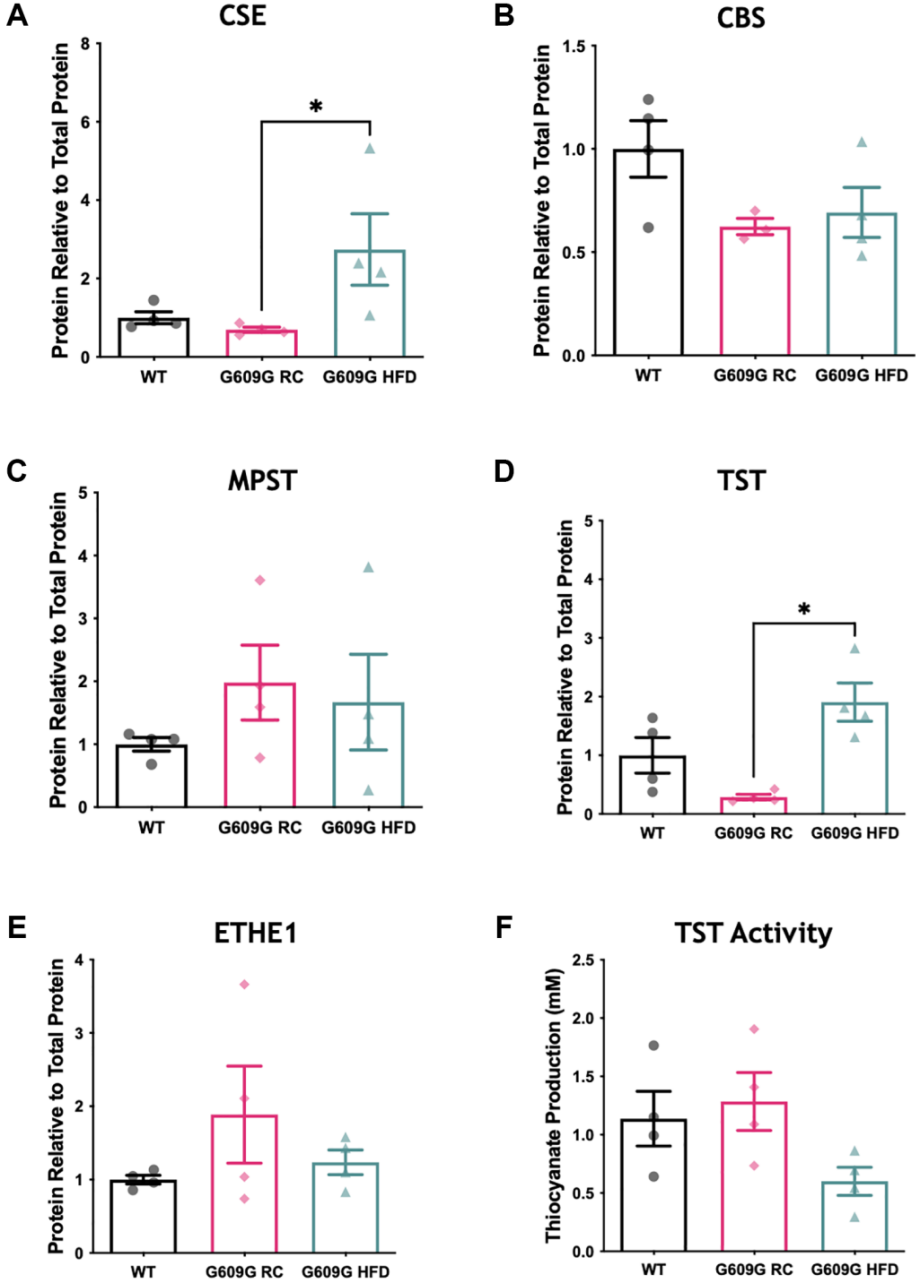
**Hepatic protein levels of H_2_S producing enzymes.** Western blotting of hepatic protein lysates quantified by densitometry analysis and expressed relative to total protein loading for (**A**) CSE, (**B**) CBS, and (**C**) MPST (**D**) TST (**E**) ETHE1. (**F**) Activity of TST protein in hepatic protein lysates as measured by a thiocyanate production capacity assay. 20 ug of liver protein lysates was loaded per well in triplicate WT data shown in black, G609G RC in pink, G609G HFD shown in green. Statistical significance was determined by Kruskal-Wallis non-parametric ANOVA test with alpha = 0.05, with Dunn's correction for multiple comparison. Grubbs outlier test with alpha = 0.05 was performed, no outliers removed. Bars show mean values with error bars representing standard error of the mean (SEM). ^*^p < 0.05. Abbreviations: CSE: Cystathionine-beta-lyase; CBS: Cystathionine-Beta-synthase; MPST: 3-Mercaptopyruvate Sulfurtransferase; ETHE1: Ethylmalonic encephalopathy 1 protein; TST: Thiosulfate Sulfurtransferase.

The protein levels of major H_2_S producing and disposal enzymes were also measured (Figure 2, representative blots in Supplementary Figure 1). A significant difference between groups in CSE protein levels was found (H = 8.115, *p* = 0.003), Figure 2A. Dunn’s multiple comparisons determined a significant reduction of approximately 60% in mean CSE protein levels between G609G RC and G609G HFD samples (*p* = 0.013) but no significant difference between any other comparison. Neither CBS (H = 3.598, *p* = 0.178) nor MPST (H = 2.192, *p* = 0.370) differed between groups as shown in Figure 2B and Figure 2C. As with CSE, protein levels of TST (Figure 2D) were found to be significantly different between groups (H = 8.115, *p* = 0.003), with *post hoc* testing determining a significant difference between G609G RC and G609G HFD mice (*p* = 0.013) only. ETHE1 levels (Figure 2E) did not differ between groups (H = 0.731, *p* = 0.746).

Gene expression analysis showed that TST mRNA transcripts were significantly reduced in G609G mice fed either diet compared to WT controls (Figure 1C). However, at the protein level there was a divergence in levels of TST protein between G609G groups, with TST significantly elevated by approximately 400% in G609G HFD compared to G609G RC (Figure 2D). We then employed a TST activity assay to interrogate the effect of genotype and diet on the rhodanese activity of TST (Figure 2F). Statistical analysis determined no significant difference between groups (H = 5.115, *p* = 0.074).

## DISCUSSION

Elevated endogenous hydrogen sulfide (H_2_S) appears to be essential for dietary restriction benefits in mice [20, 21, 30], is a conserved phenotype in long-lived mouse models [20, 21], while tissue H_2_S levels decrease with age across model organisms [31, 32]. Further, a reduction in persulfidation (a post-translational modification of cysteine residues by H_2_S) with age is a conserved phenotype across *E. coli, C. elegans, D. melanogaster,* and mice [33]. To date no studies have directly measured H_2_S production in Hutchinson-Gilford Progeria Syndrome (HGPS) despite the emerging understanding that H_2_S may be central to longevity and healthy ageing, and the commonalities seen in the signalling modalities of H_2_S and several established HGPS treatments. In this study, we employed the HGPS mouse model G609G [34] to test the hypothesis that, in contrast to anti-ageing increases in H_2_S production, the accelerated ageing typical of progeroid mice is associated with reduced hepatic H_2_S production. Given that G609G mice show lifespan extension on high-fat diet (HFD) compared to regular chow (RC) [15], we also examined the H_2_S pathway in these mice under HFD. HFD in WT mice typically shortens lifespan, and has been shown to reduce both plasma H_2_S levels and hepatic activity of CSE, CBS and MPST [26].

We report a significant reduction in H_2_S production capacity in G609G mice on RC compared to WT controls and a non-significant reduction in G609G HFD compared to WT controls, indicative of a partial rescue of H_2_S levels on HFD compared to RC in G609G mice. Cystathionine-γ-lyase (CSE) is the major producer of H_2_S in hepatic tissues [35], and transcripts for *Cse* were significantly elevated in G609G mice on both RC and HFD compared to WT controls. These data may point to a futile compensatory elevation of H_2_S producing enzymes in response to reduced H_2_S levels, as previously reported in a rat model of human myocardial infarction [36]. One potential explanation for these findings is that transcriptional regulation of *Cse* expression can be altered by reactive oxygen species (ROS). ROS, and hydrogen peroxide in particular, are capable of elevating expression of *Cse* [37]. Crucially, G609G mice on RC show chronic elevated ROS production [38]. *Cse* and *Cbs* comprise the cytosolic endogenous H_2_S production pathway in mammals, although there is also a distinct pathway for H_2_S generation within mitochondria mediated by 3-mercaptopyruvate sulfurtransferase (Mpst). However, *Mpst* transcripts were not found to be elevated in G609G RC and were in fact reduced in G609G on HFD. In order to provide additional insight, future studies could include additional dietary interventions, for example increasing or decreasing the abundance of dietary cysteine or methionine, to better understand which specific dietary components result in modification of hepatic H_2_S production in the context of the G609G mutation.

To gain better understanding of the regulation of H_2_S production we then quantified protein levels of H_2_S producing and disposal enzymes. CSE protein levels may be controlled at the transcriptional level by repression of *Cse* gene expression [21]. However, such an explanation does not account for the data presented here, where *Cse* mRNA transcripts were significantly upregulated in G609G mice on both diets but protein abundance of CSE appeared diet-dependent, being elevated in G609G mice only under HFD feeding. One potential explanation is that the extent of post-translational degradation of CSE differed between groups. Ubiquitination is an established mechanism by which CSE stability is controlled and CSE ubiquitination and degradation is promoted by superoxide anions [39]. Indeed, the difference between RC and HFD-fed G609G mice in CSE protein levels could also be explained by the observation that, unlike most oxidative damage compounds, superoxide anion generation in rodent liver is reduced by HFD [40]. As such, future studies should measure ROS production (e.g., superoxide anion), SOD activity, and/or markers of oxidative damage as these may help understand both the transcriptional and post-translational regulation of CSE. While HFD in WT mice has deleterious effects on H_2_S metabolism and nutrient metabolism [26], in G609G mice it appears to be beneficial to lifespan [15]. This has implications for the importance of nutrient rich diets in human patients with HGPS, where “nutritious and high calorie foods and supplements” are recommended [41]. We also found that TST protein was elevated in HFD-fed G609G mice compared to RC fed animals, but the H_2_S-oxidising activity of this enzyme was decreased under HFD. We previously identified TST as a putative antidiabetic enzyme and demonstrated that homozygous TST knock out confers a pro-diabetogenic HFD-like phenotype in C57BL/6J mice [42]. These findings indicate an antagonistic relationship between HFD and TST activity, which might explain our observation of reduced TST activity under HFD feeding in G609G mice.

## CONCLUSIONS

This study was designed and undertaken due to the lack of understanding in the mechanistic targets of known treatments against HGPS and considering the positive association between H_2_S and longevity in model organisms. The data acquired here confirmed some aspects of the relevance of H_2_S in HGPS but is of course descriptive in nature and raises more questions about the specific mechanisms at play. Studies in primary cell culture from patients and progeria animal models that knock down/out or overexpress *CSE* would assist in proving a mechanistic role for H_2_S in the first instance. Lentiviral constructs for the overexpression of progerin in mammalian cells are already established (see [43]) and could help clarify any direct relationship between progerin protein levels and H_2_S production. Subsequent application of H_2_S donating compounds (such as NaHS and GYY4137 [44]) could then be used to investigate if exogenous H_2_S treatment can rescue the progeroid phenotype, establish causation and a potential therapeutic window. Supplementation or depletion of cysteine in the diets fed to G609G mice would also be a key experiment to demonstrate a nutritional basis for modulating tissue levels of various sulfide species, including H_2_S, in the context of progeria. Additionally, sex differences play an important role in the regulation of *Cse* expression and in mammalian lifespan, with oestrogen, for example, acting as a positive regulator of *Cse* expression [45]. Future studies could include sex-specific control groups to untangle the role of sex on H_2_S metabolism in progeria. Finally, additional limitations in this study were a lack of statistical power due to the small sample size reported and that we were limited to a single tissue, the liver. This was due to the difficulty in breeding and maintenance of mice with the G609G mutation [34]. These mice are infertile, have an abundance of comorbidities, and due to their small size have limited tissue available. Regardless, the work presented here addresses an area of research that remains critically understudied and provides new evidence that the accelerated ageing phenotype observed in HGPS may be partially explained by a reduction in hepatic H_2_S levels.

## MATERIALS AND METHODS

### Animal model, high-fat diet, and experimental design

Mice carrying the human HGPS single-base mutation (LMNA^G609G−/+^) were generated on a C57BL/6 background by Prof. Carlos López-Otin (Universidad de Oviedo, Spain) [10]. Heterozygous LMNA^G609G−/+^ female and male mice were obtained from the Otin lab and subsequently bred in the laboratory of Prof. Susana Gonzalo (Saint Louis University, MO, USA) to generate wild type (WT) control mice and homozygous LMNA^G609G+/G609G+^ mice, referred to simply as G609G mice hereafter [15]. All animal studies were approved (protocol #2299) and conducted in accordance with the Animal Studies Committee at Saint Louis University. Mice were housed at a constant temperature of 23°C with food and water provided *ad libitum* under a 12:12 light-dark cycle. Litter mates of G609G genotype were randomly assigned into one of two diet groups and fed either regular chow (RC) or high fat diet (HFD) immediately post-weaning (approximately 3 weeks of age)- diet composition for diets is provided in Table 1. Mice were culled by cervical dislocation at either 70 days (WT, males, *n* = 4), 100 days (G609G RC, females, *n* = 4) or 150 days (G609G HFD, females, *n* = 4). Immediately following death, the liver was collected, snap-frozen in liquid nitrogen and stored at −80°C. Tissue samples were subsequently shipped to University of Glasgow for analysis.

**Table 1:** Composition of regular chow and high fat diet [25].

	**Regular Chow (RC)**	**High fat diet (HFD)**
**Supplier**	LabDiet	ResearchDiets
**Diet Reference Number**	5053	D12492
**kcal/g**	3	5.2
**Protein (% kcal)**	24.5	20
**Carbohydrates (% kcal)**	62.4	20
**Fat (% kcal)**	13.1	60

### Hepatic hydrogen sulfide (H_2_S) production capacity

Measurement of H_2_S levels was performed in liver homogenates according to the previously described method [46]. Briefly, 100 mg of flash-frozen liver tissue was lysed in passive lysis buffer. Protein concentration was determined by bicinchoninic acid (BCA) assay (G Biosciences, MO, USA) and 100 ug of protein was loaded onto a 96-well plate. 150 uL of reaction solution containing 10 mM L-cysteine and 1 mM pyridoxal-5′-phosphate was added to the protein. Filter paper that had previously been cut to the size of the plate, soaked in 20 mM lead (II)acetate trihydrate for 20 min, then dried under vacuum, was then securely attached to the plate. The assembled plate was incubated at 37°C for 1 hr. H_2_S sulfide gas produced during this time collects in the head space between the top of the solution in the well and the lead (II)acetate paper, forming a brown-black substrate on the paper. The amount of H_2_S generated in each sample was quantified by densitometry analysis of the brown-black substrate (ImageJ).

### Reverse transcriptase quantitative-PCR

RNA was isolated from liver tissue by addition of 500 μL TRIzol (Life Technologies, NY, USA) to sections of liver tissue and homogenized using a glass-glass homogeniser. Samples were moved to screw top Eppendorf and 150 uL chloroform added. Samples were then spun by centrifuge at over 8000 g and the supernatant containing the RNA isolate was taken to a fresh Eppendorf. RNA clean-up was performed using a RNAeasy Mini Kit (Qiagen, Germany), including the optional DNase digestion step. First strand synthesis of cDNA was performed by incubating 2 ug of RNA (quantified by spectrophotometry using Nanodrop 1000 UV-Vis spectrophotometer, Thermo Fisher Scientific, MA, USA) with 0.333 uL 3 ug/uL Random Primer Mix (Invitrogen, MA, USA) in a total volume of 15 uL with RNAse-free water at 70°C for 5 min using a MJ research PTC-200 Peltier Thermal Cycler (BioRad, CA, USA). Synthesis of cDNA was then performed by adding 10 uL master mix (1 uL Promega M-MLV reverse transcriptase, 5 uL Promega M-MLV 5× buffer, 5 uL pooled 10 mM dNTPs, and 0.625 uL RNAseOUT 40 units/uL) to the first stand sample and heating to 37°C for 1 hour. Samples were then diluted 1:1 with PCR-grade water and used directly for RT-qPCR. RT-qPCR was performed in a 384-well PCR plate. Each well contained 1 ul cDNA, 0.25 uL 10 mM upper primer, 0.25 uL 10 mM lower primer, 3.5 uL PCR-grade water and 5 uL QuantiFast SYBR green PCR master mix (Qiagen, UK).

The PCR reaction was performed using a 7900HT Fast Real-Time PCR System (Applied Biosystems, CA, USA) using the following conditions: 95°C for 5 minutes; 94°C for 30 seconds, 60°C for 30 seconds, 72°C for 30 seconds for 40 cycles; 72°C for 5 mins. Fold change was calculated following the ΔΔCt method and expressed as 2^−ΔΔCt^. The endogenous control gene was β2M, which has been previously shown to be an appropriate housekeeping control gene for studies that alter diet [47]. Primers were designed using the UPL Library Assay Design Centre (Roche, Switzerland) and BLASTn (NCBI) and primer sequences are provided in Table 2.

**Table 2:** Primer sequences used in RT-qPCR experiments.

**Gene name**	**Amplicon (nt)**	**Length of intron(s) spanned (nt)**	**Forward primer sequence**	**Reverse primer sequence**
CBS	84	903	gctgggcacactctctcac	caggcctggtctcgtgat
CSE	78	985	catgctaaggccttcctcaa	ctcagccagactctcatatcctc
TST	82	5208	ccagctggtggactctcg	gtggcccgagtctagtcct
MPST	85	2772	cttgccgagtgccttcac	gcctaggagatgctcagattg
ETHE1	88	11056	gattccatccgctttggac	ggtcgttcaggacaaaggtg
SUOX	131	257	ttccacaggccatcagagt	ccatctccgagtccttgagt
β2M	75	−	acagttccacccgcctcacatt	tagaaagaccagtccttgctgaag

Abbreviations: CBS: Cystathionine-β-synthase; CSE: Cystathionine-γ-lyase; TST: Thiosulfate Sulfurtransferase; MPST: 3-Mercaptopyruvate Sulfurtransferase; ETHE1: Ethylmalonic encephalopathy 1 protein; SUOX: Sulfite Oxidase; β2M: beta-2 microglobulin.

### Western blotting

Protein lysate was obtained by homogenisation of liver tissue in 1 mL ice cold RIPA buffer (Radio Immunoprecipitation Assay Buffer; 150 mM sodium chloride, 1% NP-40 or Triton X-100, 0.5% sodium deoxycholate, 0.1% sodium dodecyl sulphate, 50 mM Tris, pH 8) containing protease and phosphatase inhibitors (Halt™ Protease and Phosphatase Inhibitor Cocktail, Thermo Fisher Scientific, UK; phenylmethylsulfonyl fluoride, Sigma Life Sciences, Germany; complete Mini EDTA-free protease inhibitor cocktail, Merck, NJ, USA) using a glass-glass homogeniser. Homogenate was left on ice for 40 mins and then spun by centrifuge at over 8000 g for 10 minutes at 4°C. The supernatant was subsequently collected and used as protein lysate. Protein concentration was assessed by BCA assay (G Biosciences, MO, USA) and 20 ug of protein was loaded per well into homemade 4–12% bis-tris polyacrylamide gels. Precision Plus Protein™ Dual Xtra Standards protein marker (BioRad, CA, USA) were added to a well on each gel. Proteins were separated by electrophoresis at 90 V for 90 mins and then transferred onto nitrocellulose membrane at 0.25 V for 1 hour. Membranes were stained with Ponceau-S (Sigma Life Sciences, Germany), briefly washed in deionised water and the resulting total protein stain was captured using a Chemidoc™ XRS System (BioRad, CA, USA). The Ponceau-S stain was removed by 1xTBST (Tris-Buffered Saline Tween^20^) and the membrane was blocked with 5% milk in 1xTBST for 40 min. The membrane was washed 5 times with 1xTBST for 5 mins under constant shaking. Primary antibodies (Abcam, Cambridge, UK) were added to the membrane in 5% BSA in 1xTBST. Cystathionine-γ-lyase (CSE, ab151769) and ethylmalonic encephalopathy 1 protein (ETHE1, ab174302) primary antibodies were used at 1:1000 dilution, cystathionine-β-synthase (CBS, ab135626), 3-mercaptopyruvate sulfurtransferase (MPST, ab85377), and thiosulfate sulfurtransferase (TST, ab155320) were used at 1:500 dilution and total OXPHOS rodent cocktail (ab110413) was used at 1:10000 dilution. Primary antibodies were allowed to incubate with the membrane overnight at 4°C, under constant shaking. Anti-rabbit secondary antibody (Anti-rabbit IgG Alexa Fluor 680 conjugate, Abcam ab186696) was used at 1:10000 dilution in 5% BSA in 1xTBST as the secondary antibody for all blots. The secondary antibody was allowed to incubate with the membrane for 1 hour at room temperature, under constant shaking. The membrane was washed 5 times with 1xTBST for 5 mins, under constant shaking before addition of all antibodies and before imaging by an Odyssey M Fluorescent imager (LiCor, NE, USA). Protein signals were quantified using densitometry software (ImageStudio; LiCor, NE, USA) and normalised to the total protein signal of their respective lane.

### TST activity assay

Thiosulfate sulfurtransferase (TST) rhodanese activity was determined by measuring thiocyanate production capacity as previously described [48]. The assay was performed in a 96-well plate, where 20 μg of protein lysate was added. The reaction was initiated by the addition of 10 μL 500 mM thiosulfate and taken to 90 μL with 500 mM potassium phosphate pH 5.5 buffer. After a 2 min incubation at 37°C 10 μL 500 mM potassium cyanide was added to each sample. A standard curve of 0.1, 0.25, 0.5, 1, 2.5, 5, 10, 25, 50 mM potassium thiocyanate solutions was also prepared following the same conditions as above but without the addition of potassium cyanide. The reaction proceeded for 5 min at 37°C and was stopped by the addition of 11 μL of 38% formaldehyde. Lastly, 125 μL Fe(NO_3_)_3_/26% HNO_3_ was added which produced a colorimetric change. Results were quantified by spectrophotometry, measured at 460 nm absorbance. All samples were performed in duplicate, calculating the average absorbance.

### Statistical analysis

All statistical analyses were performed using Prism 9 (GraphPad Inc., La Jolla, CA, USA) software. Data were first analysed by Grubbs outlier test with alpha set to 5%. Any outliers identified are noted in figure legends. Statistical significance was determined by performing non-parametric Kruskal-Wallis tests comparing WT, G609G RC and G609G HFD groups with Dunn’s correction for *post-hoc* multiple comparison tests. ^*^ denotes a *p* value of < 0.05, ^**^ denotes a *p* value of < 0.01, and ^***^ denotes a *p* value < 0.001.

## Supplementary Materials

Supplementary Figure 1
