# Supplementary material for: Hepatic hydrogen sulfide levels are reduced in mouse model of Hutchinson-Gilford progeria syndrome
Source: Aging (Albany NY). 2023 Jun 23;15(12):5266–78. doi: 10.18632/aging.204835 (PMC10333079; doi:10.18632/aging.204835)
Supplement: Supplementary Figure 1 [file aging-15-204835-s001.pdf]

SUPPLEMENTARY FIGURE

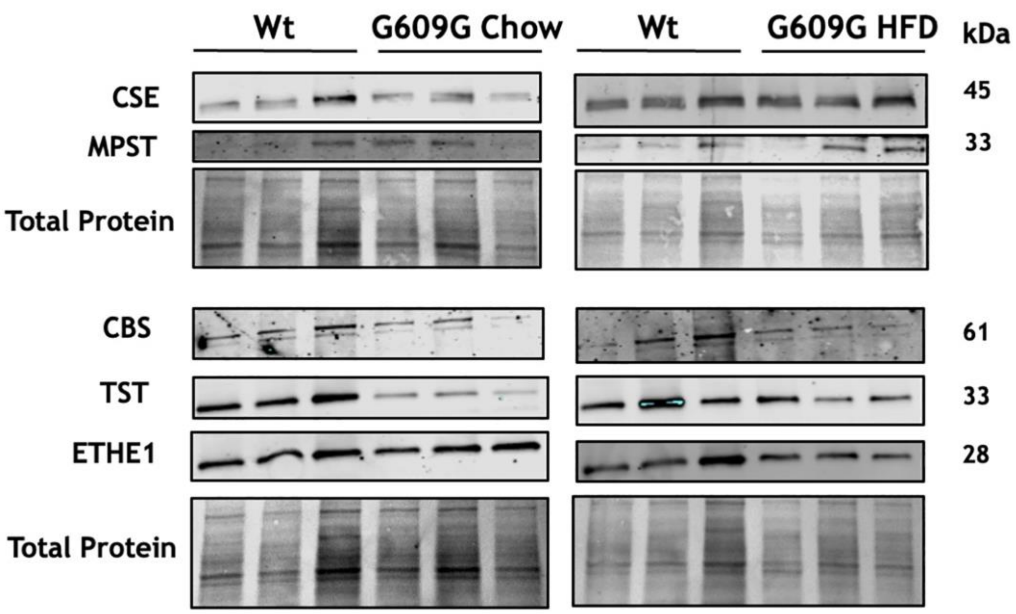

Supplementary Figure 1. Representative western blot images. Representative western blot images, three replicates per group. Total protein image acquired by staining blot in ponceau S stain prior to blocking of membrane. Abbreviations: CSE: Cystathionine-beta-lyase; CBS: Cystathionine-Beta-synthase; MPST: 3-Mercaptopyruvate Sulfurtransferase; ETHE1: Ethylmalonic encephalopathy 1 protein; TST: Thiosulfate Sulfurtransferase.
